# Results of a global, patient-based survey assessing the impact of psoriatic arthritis discussed in the context of the Psoriatic Arthritis Impact of Disease (PsAID) questionnaire

**DOI:** 10.1186/s12955-020-01422-z

**Published:** 2020-06-08

**Authors:** L. C. Coates, A.-M. Orbai, V. F. Azevedo, J. C. Cappelleri, K. Steinberg, R. Lippe, I. Lim, L. Eder, P. Richette, M. Y. Weng, R. Queiro Silva, L. Fallon

**Affiliations:** 1 Nuffield Department of Orthopaedics, Rheumatology and Musculoskeletal Sciences, Botnar Research Centre, University of Oxford, Windmill Road, Oxford, OX3 7LD UK; 2 Johns Hopkins University School of Medicine, Baltimore, MD USA; 3 Universidade Federal do Paraná, Curitiba, Brazil; 4 Pfizer Inc, Groton, CT USA; 5 The Harris Poll, Rochester, NY USA; 6 Pfizer Pharma GmbH, Berlin, Germany; 7 BJC Health, Sydney, NSW Australia; 8 Women’s College Research Institute, University of Toronto, Toronto, ON Canada; 9 Lariboisière Hospital, Lariboisière, University of Paris 7, Paris, France; 10 Department of Internal Medicine, Division of Allergy, Immunology and Rheumatology, National Cheng Kung University Medical College and Hospital, Tainan, Taiwan; 11 Rheumatology Division, HUCA, Oviedo, Spain; 12 Pfizer Inc, Montreal, QC Canada

**Keywords:** Global survey, Patient-reported outcomes, Population-based study, Psoriatic arthritis

## Abstract

**Background:**

Psoriatic arthritis (PsA) is a chronic immune-mediated inflammatory musculoskeletal disease, manifesting as peripheral arthritis, enthesitis, dactylitis, spondylitis, and skin and nail psoriasis. A core set of domains for measuring the impact of PsA has been developed, including pain, patient global assessment, physical function, health-related quality of life (HRQoL), and fatigue. To understand the impact of PsA on health domains from a patient’s perspective, a global survey was developed and results reported in the context of the 12-item Psoriatic Arthritis Impact of Disease (PsAID-12) questionnaire.

**Methods:**

An online patient-based global survey was conducted by The Harris Poll in Australia, Brazil, Canada, France, Spain, Taiwan, the UK, and the US between November 2, 2017 and March 12, 2018. Eligible patients were ≥ 18 years old with a diagnosis of PsA for > 1 year, had visited a rheumatologist/dermatologist in the past 12 months and reported using ≥ 1 synthetic/biologic disease-modifying antirheumatic drug for PsA. Patients reported on PsA severity and symptoms, and the impact of PsA on HRQoL. After survey completion, responses were aligned with PsAID health domains. Descriptive statistics and chi-square tests were conducted.

**Results:**

This analysis included 1286 patients from eight countries. Most patients (97%) reported musculoskeletal symptoms relating to PsA in the past year. Common moderate/major impacts of PsA were on physical activity (78%), ability to perform certain activities (76%), work productivity (62%), and career path (57%). Skin/nail symptoms occurred in 80% of patients. Overall, 69% of patients reported that PsA had a moderate/major impact on emotional/mental wellbeing, 56% on romantic relationships/intimacy, and 44% on relationships with family and friends. Social impacts included emotional distress (58%), social shame or disapproval (32%), and ceased participation in social activities (45%). Over half of all patients experienced unusual fatigue over the past 12 months (52%). The health domains that patients reported as being impacted by PsA aligned with life impact domains of the patient-derived PsAID health domains.

**Conclusion:**

These results highlight the impact of PsA on multiple health domains from a patient perspective that should be considered during shared decision-making processes between healthcare providers and patients.

## Background

Psoriatic arthritis (PsA) is a chronic, immune-mediated, inflammatory musculoskeletal disease, manifesting as peripheral arthritis, enthesitis, dactylitis, spondylitis, and skin and nail psoriasis [1, 2]. The prevalence of PsA has been reported as ranging from 0.01 to 1% depending on geographic region [3–5] and it has been estimated that around 6–42% of patients with psoriasis develop PsA [3, 4, 6, 7]. Patients are commonly diagnosed with psoriasis around 10 years before the onset of musculoskeletal symptoms [4]. The symptomatic burden of PsA can negatively impact physical and mental health, as well as daily activities, sleep, work and leisure activities, and social participation, resulting in a reduced health-related quality of life (HRQoL) [8–11].

In order to appropriately evaluate the efficacy of new therapies in clinical trials of PsA, it is important to identify measures that can assess outcomes of relevance to both patients and physicians [12]. Such measures should reflect the core set of domains proposed by The Group for Research and Assessment of Psoriasis and Psoriatic Arthritis (GRAPPA) - Outcome Measures in Rheumatology (OMERACT) working group for evaluating the impact of PsA in clinical trials, and include pain, patient global assessment, physical function, HRQoL, and fatigue [13].

The PsA Impact of Disease (PsAID) questionnaire was developed by a European League Against Rheumatism (EULAR) taskforce including patients and clinical experts from 13 countries, with the aim of providing an instrument that assesses the impact of PsA from the patients’ perspective. The PsAID-12 consists of 12 items, each representing a PsA-specific life-impact domain: pain, fatigue, skin problems, work and/or leisure activities, functional capacity, discomfort, sleep disturbance, coping, anxiety, embarrassment and/or shame, social participation, and depression [14]. A shorter version of the PsAID-12 questionnaire, the PsAID-9, is also available [14]. The domains of health assessed in the PsAID-12 have been grouped into three categories of impact: physical impact (predominantly related to joints); impact related to skin; and psychological and social impact [15]. PsAID-12 has been provisionally endorsed as a core outcome measure for disease-specific HRQoL in PsA clinical trials [16].

The PsAID-12 has been shown to have high content validity for patients [14, 17]. In a study to assess the construct validity, reliability, and interpretability of the PsAID-12, correlations were observed between PsAID scores and patients’ disease state, assessed using the clinical Disease Activity index for PSoriatic Arthritis (cDAPSA) [18]. Furthermore, in a cross-sectional multicenter study conducted in Spain, PsA was observed to have significantly lower impact in patients with minimal disease activity (MDA) than patients not in a state of MDA for all domains of the PsAID [19], which supports the relevance of the domains assessed for patients with PsA.

A real-world, anonymized PsA narrative global patient survey was developed by The Harris Poll to further evaluate the impact of PsA on daily life from the patients’ perspective, to provide global and country-specific evaluations, and to support the relevance of using PsAID in clinical practice. The survey was specific to patients with PsA, and spanned topics including psoriasis experience, quality of life and general feelings towards PsA, experience pre-diagnosis and at initial diagnosis, relationships and communication with physicians, treatment attitudes and experiences, and feelings towards changes in medication regimens. Survey questions were collated and analyzed post-hoc to align, in part, with validated PsAID categories of impact, and here we report the results of this analysis.

## Methods

### Study design and patients

Data were collected via an online patient survey, conducted by The Harris Poll in Australia, Brazil, Canada, France, Spain, Taiwan, the UK, and the US from November 2, 2017 to March 12, 2018.

Patients were recruited from online market research panels made up of members who agreed to participate in this type of research. Prospective respondents participating through online panels were sent an invitation with a link to the online survey. Once respondents had entered the survey, completed the survey screener, and were identified as a qualified respondent with PsA, they were required to provide consent to continue to the core survey content. Each country had a custom set of demographic questions. Patients answered between approximately 40–60 questions depending on their country and answers to previous questions.

Eligible patients were ≥ 18 years old with a self-reported diagnosis of PsA for > 1 year prior to participation in the survey. Patients were required to have visited a rheumatologist or dermatologist in the past 12 months and reported ever using ≥ 1 synthetic or biologic disease-modifying antirheumatic drug (DMARD) for PsA. All participants provided informed consent to complete the research.

Estimated qualification rates were calculated based on the approximate total number of individuals who entered the survey versus the final qualified completers.

### Endpoints and analysis

The survey included questions related to psoriasis experience, quality of life and general feelings towards PsA, experience pre-diagnosis and at initial diagnosis, treatment attitudes and experiences, and feelings towards changes in medication regimens. A subset of survey questions that most closely aligned with the PsAID questionnaire in terms of content and wording were selected for analysis (Supplementary Table 1). These specific questions assessed overall health, PsA severity, PsA symptoms, and the impact of PsA on HRQoL, including physical function, work, social life, and emotional health. Questions were categorized into domains of health to further assess the effect of PsA on the daily lives of patients (Supplementary Table 2).

### Statistical analysis

Analyses included descriptive statistics and also binomial (chi-square) tests for two proportions [20, 21]. Raw data were not weighted at the individual country level and are therefore only representative of the individuals who completed the survey. The unweighted sample sizes reflect the total number of patients who completed the survey. For the global, eight-country total, a post-weight was applied to adjust for the relative size of each country’s adult population within the total adult population across all countries surveyed. All reported percentages were calculated and analysed based on the weighted global total. For statistical testing, the effective base was used, which reflects the effective sample size (and hence precision due to weighting) and aims to reduce the impact of dramatic weighting on the outcome for statistical testing [21]. Statistical testing was performed to compare differences between countries. Statistical significance was defined as *p* < 0.05.

## Results

### Patients

A total of 1286 patients from eight countries responded to the survey, met the eligibility criteria, and were included in the analysis. The total estimated qualification rate was 38% (1286/3405) and ranged from 26 to 57% in individual countries.

Demographics and disease characteristics are shown in Table 1. In the total global population, 52% of respondents were female and the mean age was 41 years. There was a significant difference in age between Brazil (33.4 years) and all other countries (range 36.9–49.1 years; *p* < 0.05) and the proportion of female patients was significantly higher in the US (61%) compared with all other countries, except Canada (52%) (range in other countries 40–52%; *p* < 0.05) (Table 1).

**Table 1:** Demographics and disease characteristics by country

	Global total	Australia	Brazil	Canada	France	Spain	Taiwan	UK	US
Unweighted Base, N	1286	152	152	155	123	135	109	159	301
Weighted Base, N	1286	39	319	62	111	85	41	109	521
Age, years, mean (SD)	41.2 (13.3)	45.7 (13.7)	33.4 (8.9)	49.1 (15.4)	40.9 (12.6)	36.9 (9.1)	43.3 (10.4)	41.5 (11.5)	45.1 (14.2)
Female, n (%)	674 (52)	16 (41)	151 (47)	32 (52)	49 (44)	35 (41)	17 (40)	56 (52)	318 (61)
Current overall health, n (%)^a^									
Excellent	57 (4)	1 (2)	4 (1)	3 (5)	3 (2)	1 (1)	2 (4)	2 (2)	42 (8)
Good	388 (30)	10 (27)	94 (30)	27 (45)	13 (12)	5 (6)	6 (15)	19 (17)	213 (41)
Fair	674 (52)	21 (55)	187 (59)	26 (42)	74 (67)	42 (50)	26 (63)	67 (61)	232 (45)
Poor	167 (13)	6 (16)	34 (11)	5 (8)	21 (19)	37 (44)	8 (18)	22 (20)	35 (7)
Time since diagnosis of PsA, years, mean (SD)	9.0 (8.6)	8.5 (8.2)	7.2 (6.0)	12.3 (11.4)	8.5 (6.3)	8.8 (7.2)	6.2 (5.0)	8.1 (6.3)	10.3 (10.5)
Current PsA disease severity, n (%)^b^									
Mild	205 (16)	11 (28)	50 (16)	22 (35)	12 (11)	13 (15)	17 (40)	22 (20)	59 (11)
Moderate	849 (66)	24 (61)	243 (76)	32 (52)	75 (67)	67 (79)	21 (51)	73 (67)	315 (60)
Severe	232 (18)	4 (11)	25 (8)	8 (12)	24 (22)	6 (7)	3 (8)	14 (13)	147 (28)
Current PsA medication, n (%)									
bDMARD only	483 (38)	7 (19)	78 (24)	23 (38)	50 (46)	20 (24)	9 (22)	25 (23)	270 (52)
Oral DMARD only	419 (33)	14 (36)	120 (37)	18 (30)	31 (28)	41 (49)	20 (49)	45 (42)	130 (25)
Oral DMARD + bDMARD	228 (18)	6 (16)	80 (25)	11 (17)	13 (11)	14 (17)	9 (23)	15 (14)	80 (15)
NSAIDs/steroids only	140 (11)	10 (27)	42 (13)	8 (13)	14 (13)	8 (10)	3 (6)	23 (21)	31 (6)
Not sure	6 (< 0.5)	< 0.5 (1)	0 (0)	1 (1)	3 (2)	1 (1)	0 (0)	0 (0)	2 (< 0.5)
Not currently taking PsA medication	10 (1)	1 (1)	0 (0)	< 0.5 (1)	0 (0)	0 (0)	0 (0)	0 (0)	9 (2)

Raw data were not weighted at the individual country level, and are therefore only representative of the individuals who completed the survey. For the global, eight-country total, a post-weight was applied to adjust for the relative size of each country’s adult population within the total adult population across all countries surveyed. The unweighted sample sizes reflect the total number of patients who completed the survey, while all reported percentages are calculated based on the weighted global total as the denominator. Percentages might not exactly match those derived by manual calculation due to weighting and/or computer rounding

*bDMARD* Biologic disease-modifying antirheumatic drug, *NSAID* Non-steroidal anti-inflammatory drug, *PsA* Psoriatic arthritis, *SD* Standard deviation

^a^Patients were asked, “How would you describe your current overall health today?” and gave subjective answers of either “excellent”, “good”, “fair”, or “poor”, without formal definition of the designations

^b^Patients were asked, “How bad is your psoriatic arthritis today?” and gave subjective answers of either “mild”, “moderate”, or “severe”, without formal definition of the designations

Overall, nearly two-thirds of patients described their current overall health as poor/fair (65%), while over one-third described it as excellent/good (35%). The majority of patients (84% overall) reported moderate/severe PsA. A significantly greater proportion of patients in Canada (35%) and Taiwan (40%) reported mild disease versus other countries (range 11–20%; *p* < 0.05) except Australia (28%) (Table 1). Mild, moderate, and severe designations of PsA were subjectively reported by each patient.

In total, nearly 88% of patients were currently taking DMARDs, and 11% were taking non-steroidal anti-inflammatory drugs (NSAIDs)/steroids for their PsA. Globally, 38% of patients were taking biologic (b)DMARDs only, 33% were taking oral DMARDs only, and 18% were taking both bDMARDs and oral DMARDs. Significantly more patients reported using bDMARDs as monotherapy in Canada (38%), France (46%), and the US (52%) versus other countries (range 19–24%; *p* < 0.05) (Table 1).

### Physical impact of PsA

**Fig. 1:**
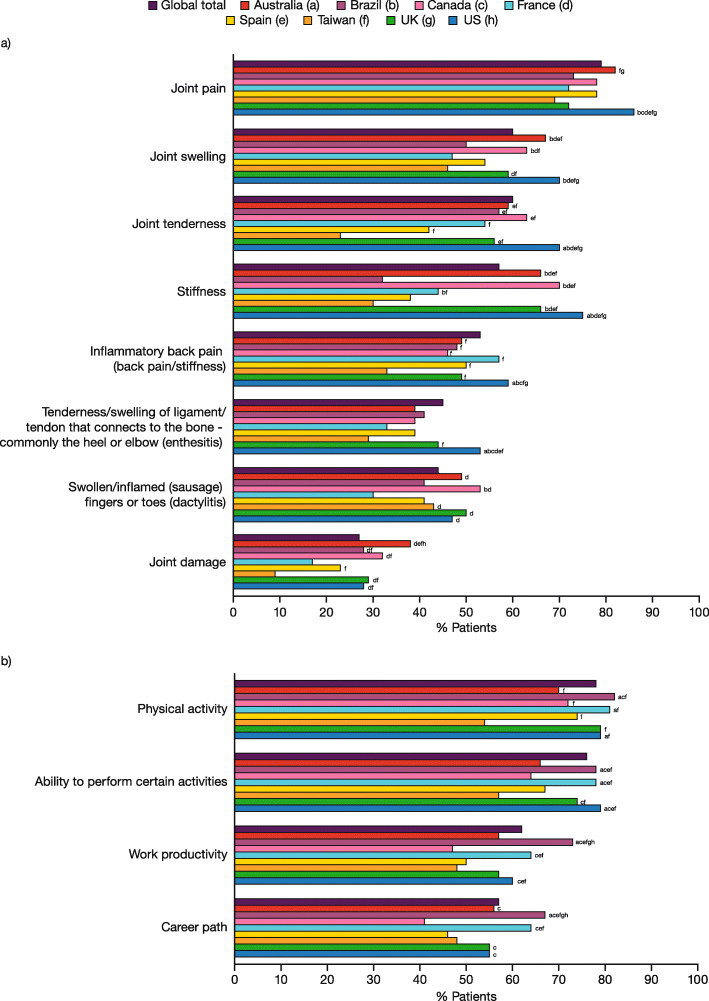
Physical impact of PsA. **a** Patient-reported musculoskeletal symptoms related to PsA experienced in the past 12 months; **b** Patient-reported moderate/major physical impact of PsA. Data represent the percentage of responders using the weighted base of each country in Table 1. **a** Data are reported in response to the question, “Which of the following symptoms, if any, have you experienced in the past 12 months related to psoriatic arthritis? Please select all that apply.” **b** Data are reported in response to the question, “How much of a negative impact, if any, has psoriatic arthritis had on each of the following aspects of your life (no impact/slight/moderate/major)?” Data represent sum of moderate/major impact, and countries are represented by colored data bars. Significant differences (*p* < 0.05) between countries are designated by the letters following the bars: a = Australia; b = Brazil; c = Canada; d = France; e = Spain; f = Taiwan; g = UK; h = US. PsA, psoriatic arthritis.

Nearly all patients surveyed (97%) reported experiencing musculoskeletal symptoms related to PsA in the 12 months prior to the survey. Joint pain, joint tenderness, and joint swelling were the most frequently reported musculoskeletal symptoms (79, 60, and 60%, respectively; Fig. 1a), which is consistent with research that led to the development of the PsAID (where pain was identified as the most prioritized domain of PsA) [14]. A significantly greater proportion of patients reporting joint pain, joint tenderness, joint swelling, stiffness, inflammatory back pain, and enthesitis were from the US compared with most other countries (Fig. 1a).

Of the 1270 patients currently taking prescription medication for their PsA, 91% reported still experiencing musculoskeletal symptoms, including 53% who reported still experiencing joint pain. Joint pain and inflammatory back pain were the musculoskeletal symptoms considered most bothersome by the greatest proportion of patients (32 and 12%, respectively), while joint tenderness, joint swelling, stiffness, enthesitis, dactylitis, and joint damage were each reported as most bothersome by ≤ 6% of patients.

Overall, 78% of patients reported a moderate/major impact of PsA on physical activity (Fig. 1b). The proportion of patients reporting a moderate/major impact of PsA on physical activity was significantly lower in Taiwan (54%) than in all other countries surveyed (range 70–82%; *p* < 0.05; Fig. 1b).

The majority of patients also reported a moderate/major impact of PsA on ability to perform certain activities (76%), work productivity (62%), and career path (57%) (Fig. 1b). A significantly greater proportion of patients in Brazil, France, and the US reported a moderate/major impact on ability to perform certain activities (range 78–79%) versus Australia, Canada, Spain, and Taiwan (range 57–67%; *p* < 0.05), as well as a moderate/major impact on work productivity (range 60–73%) versus Canada, Spain and Taiwan (range 47–50%; also versus Australia, the UK, and the US for Brazil; *p* < 0.05) (Fig. 1b). The proportion of patients reporting a moderate/major impact of PsA on career path was significantly higher in Brazil (67%) than in all other countries surveyed (range 41–56%; all *p* < 0.05), with the exception of France (64%) (Fig. 1b).

The majority of patients (81%) reported an impact of PsA on work, including, for example, having to take a sick day (49%), decreased productivity (42%), having to take medical leave (34%), switching jobs (12%), quitting or being let go from a job (13%), and permanent disability (12%) (Supplementary Figure 1).

### Impact of PsA on skin and nails

**Fig. 2:**
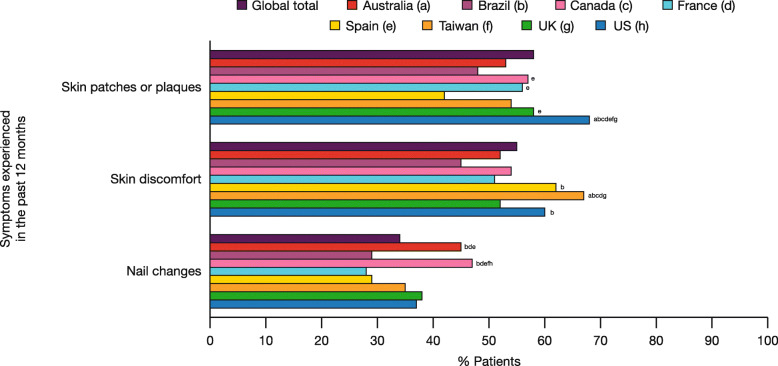
Patient-reported skin or nail symptoms. Data represent the percentage of responders using the weighted base of each country in Table 1. Data are reported in response to the question, “Which of the following symptoms, if any, have you experienced in the past 12 months related to psoriatic arthritis? Please select all that apply.” Countries are represented by colored data bars. Significant differences (*p* < 0.05) between countries are designated by the letters following the bars: a = Australia; b = Brazil; c = Canada; d = France; e = Spain; f = Taiwan; g = UK; h = US

Overall, 80% of patients reported experiencing skin and/or nail symptoms related to PsA during the past 12 months. Skin patches/plaques (e.g., flaking, redness, etc.) (58%) and skin discomfort (e.g., itching, painful, bleeding, etc.) (55%) were reported by more than half of the patients, and nail changes (e.g., pitting or small dents, separation from nail bed, etc.) by 34% (Fig. 2). A significantly higher proportion of patients in the US (68%) reported skin patches/plaques than in all other countries (range 42–58%; *p* < 0.05; Fig. 2). A higher proportion of patients in Taiwan (67%) reported skin discomfort than in Australia, Brazil, Canada, France, or the UK (range 45–54%; *p* < 0.05; Fig. 2). The highest proportions of patients reporting nail changes were in Australia (45%) and Canada (47%), which were each significantly higher than Brazil, France, and Spain (range 28–29%; *p* < 0.05) (Fig. 2). The proportion of patients reporting nail changes in Canada was also significantly higher than in Taiwan and the US (35 and 37%, respectively; *p* < 0.05) (Fig. 2).

Of patients currently taking prescription medication for their PsA, 40, 35, and 27% still experienced skin patches/plaques, skin discomfort, and nail changes, respectively. A significantly greater proportion of patients in Taiwan (53%) currently taking prescription medication for their PsA reported still experiencing skin discomfort, compared with all other countries (range 29–40%; *p* < 0.05).

### Psychological and social impact of PsA

**Fig. 3:**
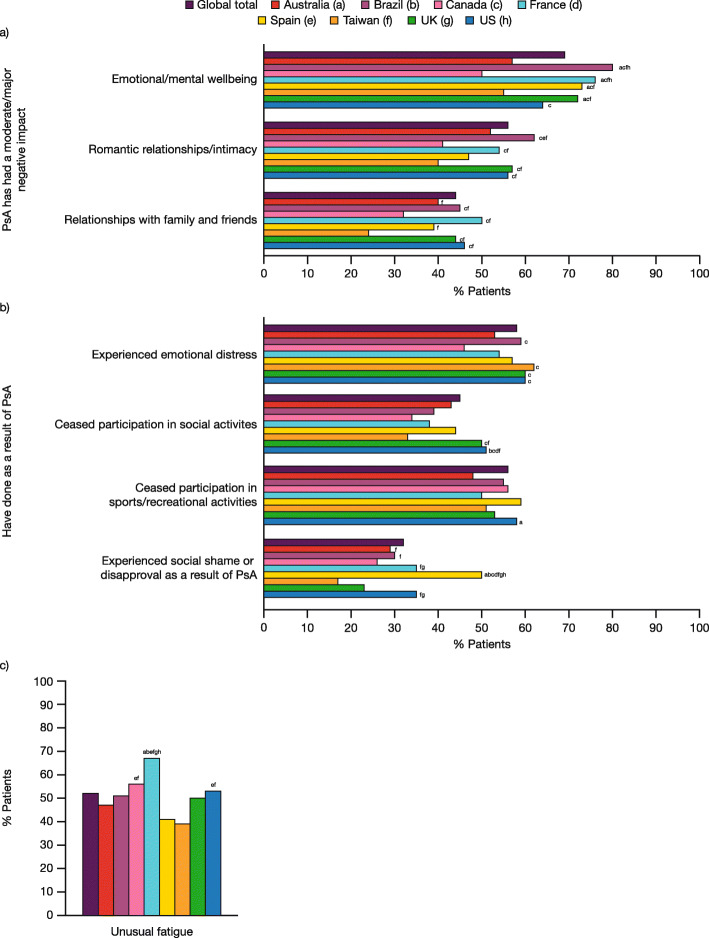
Patient-reported psychological or social impact of PsA. **a** Emotional/mental wellbeing and relationships; **b** Emotional distress and social participation; **c** Unusual fatigue. Data represent the percentage of responders using the weighted base of each country in Table 1. **a** Data are reported in response to the question, “How much of a negative impact, if any, has psoriatic arthritis had on each of the following aspects of your life (no impact/slight/moderate/major)?” Data represent sum of moderate/major impact. **b** Data are reported in response to the question, “Have you done any of the following as a result of psoriatic arthritis? Please select all that apply.” **c** Data are reported for patients selecting ‘Unusual fatigue’ in response to the question, “Which of the following symptoms, if any, have you experienced in the past 12 months related to psoriatic arthritis? Please select all that apply.” Countries are represented by colored data bars. Significant differences (*p* < 0.05) between countries are designated by the letters following the bars: a = Australia; b = Brazil; c = Canada; d = France; e = Spain; f = Taiwan; g = UK; h = US. PsA, psoriatic arthritis

Overall, 69% of patients reported that PsA had a moderate/major impact on their emotional/mental wellbeing, 56% on their romantic relationships/intimacy, and 44% on their relationships with family and friends (Fig. 3a). A significantly greater proportion of patients in Brazil, France, Spain, and the UK experienced a moderate/major negative impact on emotional/mental wellbeing (range 72–80%) compared with Australia, Canada, and Taiwan (range 50–57%; *p* < 0.05; Fig. 3a). A smaller proportion of patients in Canada and Taiwan reported a moderate/major impact on their romantic relationships/intimacy (41 and 40%, respectively) and relationships with family and friends (32 and 24%, respectively) than in other countries (romantic relationships/intimacy, range 47–62%; relationships with family and friends, range 39–50%; Fig. 3a).

Many patients reported experiencing emotional distress (58% overall), or stopping participating in social activities (45% overall) or in certain sports/recreational activities (56% overall) as a result of their PsA (Fig. 3b). The proportion of patients in Canada reporting emotional distress (46%) was significantly lower than in Brazil, Taiwan, the UK, and the US (range 59–62%; *p* < 0.05; Fig. 3b). Overall, 32% of patients had experienced social shame or disapproval as a result of their PsA, and this was more commonly reported in Spain (50%) than in other countries (range 17–35%; *p* < 0.05; Fig. 3b).

Approximately half of all patients (52%) reported experiencing unusual fatigue over the past 12 months (Fig. 3c). The highest proportion of patients reporting unusual fatigue was in France (67%); this was significantly higher than in all other countries (range 39–53%; *p* < 0.05) except Canada (56%) (Fig. 3c).

## Discussion

In this global survey of more than 1200 patients with PsA from eight countries, the majority of patients reported experiencing musculoskeletal and skin and/or nail symptoms, and that PsA impacted physical function and their ability to perform certain activities. Many patients also described a substantial impact on social, emotional, and work-related aspects of life. This was despite 99% of patients currently taking prescription medication for their PsA, with 56% receiving a bDMARD. This highlights the significant burden of PsA on HRQoL and the ongoing unmet need in this disease.

Some notable variations in responses were observed between countries. For example, musculoskeletal symptoms (specifically joint pain, joint tenderness, joint swelling, stiffness, inflammatory back pain, and enthesitis) and skin patches/plaques were more commonly reported in the US than in other countries, while social shame or disapproval was more commonly reported in Spain, and the highest proportion of patients reporting unusual fatigue was in France.

The proportions of patients in Canada reporting some musculoskeletal and skin/nail symptoms and unusual fatigue were among the highest of any country surveyed; however, this did not appear to result in higher impact being reported by Canadian patients in other health domains. For example, the proportions of patients in Canada reporting a moderate/major impact of PsA on their emotional/mental wellbeing, romantic relationships/intimacy, and relationships with family and friends were among the lowest of any participating country involved in the survey, as were the proportions of Canadian patients reporting ceased participation in social activities and experiencing emotional distress.

In Taiwan, the proportions of patients reporting a moderate/major impact of PsA on physical activity, ability to perform certain activities, work productivity, and career path were comparatively lower than in most other countries, as were the proportions reporting a moderate/major negative impact on emotional/mental wellbeing, romantic relationships/intimacy, and relationships with family and friends. Conversely, a greater proportion of patients in Taiwan reported skin discomfort compared with other countries, suggesting that skin involvement may have more of an impact in this population.

The country-specific differences observed in this study may reflect phenotypical variations in disease severity between different populations, as well as different expectations or cultural norms. Country-specific differences have also been observed in the results of a multi-national population-based survey of patients with psoriasis and PsA [22]. The findings of this survey confirm the heterogeneous nature of PsA and highlight the importance of taking into account a range of symptoms, and the related physical, psychological, and social impact when considering treatment options [23].

Given the diverse nature of aspects of daily life impacted in PsA, the use of appropriate measures to assess disease activity and additional measures to reflect the patient experience, such as PsAID, is critical [12–14]. In a study of 460 patients with PsA, it was observed that higher fatigue, lower self-perceived coping, and impaired social participation, as assessed with the PsAID, were associated with discordance between physician and patient global assessment of disease [24]. Furthermore, in a real-world study of 1201 patients with PsA from the US and Europe, active skin involvement did not influence physician global assessment scores, whereas significantly worse patient global assessment scores were observed in patients with skin involvement [25]. The result of this global survey demonstrated overall a high impact in all key PsAID domains tested, thus highlighting the usefulness of the PsAID-12 measure in routine clinical practice to improve patient–physician communication [14].

Some limitations of this study must be acknowledged. The PsAID questionnaire was not administered to patients directly; rather, an independent online survey was used, which differed from the questions and response items of the PsAID. Therefore, a correlation between the PsAID and survey responses could not be elucidated. However, findings from this comprehensive HRQoL survey were analyzed in the context of the domains of health included in the PsAID. Also, not all PsAID domains of health were captured in this survey; for example, sleep disturbance is a health domain assessed in the PsAID that was not assessed in this analysis. However, as this survey was not specifically designed to align with the PsAID, but rather to cover known impacts of PsA from the literature and to provide a global, descriptive contextualization, some additional impacts of PsA were captured here that are not covered by the PsAID, such as romantic relationships/intimacy and starting a family. Many PsAID questions assessed the impact of PsA over the preceding week, while some questions in the global narrative survey focused on PsA impact over the preceding year, which may have introduced recall bias. Although different from PsAID, a longer recall period may provide a greater breadth of information, as patients may not experience all manifestations of PsA within a given week. Additionally, data were not collected on patient-specific, non-disease-related characteristics, nor on the patients’ length of treatment. Furthermore, as this was a patient-based survey, the study was limited by patients’ abilities to understand the survey questions, to appropriately describe the diagnosis of PsA, and to accurately recall their own symptoms. The online nature of the survey may have also excluded patients without internet access or membership in online panels. Results from the survey can provide physicians with key geographical and cultural insights about how PsA impacts patients’ daily lives, and could lead to a better understanding of individual patient goals. This study is hypothesis-generating, and further research with larger patient cohorts from individual countries is needed. To reduce sample bias, respondents were recruited from online market research panels that were not specific to any disease state and the survey invite and screener were written such that the research topic remained blinded until qualification was confirmed.

## Conclusions

The results of this global survey highlight the impact of PsA on multiple health domains from a patient perspective. Notable variations in results between patients from different countries were observed and this warrants further investigation. Furthermore, the health domains that patients reported as being impacted by PsA in this survey aligned with life impact domains of the patient-derived PsAID health domains, confirming the importance of considering all potential disease impacts in shared decision making for the management of PsA. This work suggests that this PsA narrative questionnaire covers important HRQoL domains in this large sample of participants and supports the validity of the PsAID to assess disease impact in this condition.

## Supplementary information

**Additional file 1: Supplementary Table 1.** Survey questions and responses.

**Additional file 2: Supplementary Table 2.** Categorization of questions by domain of health.

**Additional file 3: Supplementary Figure 1.** Patient-reported impact of PsA on work. Data represent the percentage of responders using the weighted base of each country in Table 1. Data are reported in response to the question, “Have you done any of the following as a result of psoriatic arthritis? Please select all that apply.” Countries are represented by colored data bars. Significant differences (*p* < 0.05) between countries are designated by the letters following the bars: a = Australia; b = Brazil; c = Canada; d = France; e = Spain; f = Taiwan; g = UK; h = US. PsA, psoriatic arthritis.

## Data Availability

Upon request, and subject to certain criteria, conditions and exceptions (see https://www.pfizer.com/science/clinical-trials/trial-data-and-results for more information), Pfizer will provide access to individual de-identified participant data from Pfizer-sponsored global interventional clinical studies conducted for medicines, vaccines and medical devices (1) for indications that have been approved in the US and/or EU or (2) in programs that have been terminated (i.e., development for all indications has been discontinued). Pfizer will also consider requests for the protocol, data dictionary, and statistical analysis plan. Data may be requested from Pfizer trials 24 months after study completion. The de-identified participant data will be made available to researchers whose proposals meet the research criteria and other conditions, and for which an exception does not apply, via a secure portal. To gain access, data requestors must enter into a data access agreement with Pfizer.

## Abbreviations

bDMARD: Biologic disease-modifying antirheumatic drug
cDAPSA: Clinical Disease Activity index for PSoriatic Arthritis
DMARD: Disease-modifying antirheumatic drug
EULAR: European League Against Rheumatism
GRAPPA: The Group for Research and Assessment of Psoriasis and Psoriatic Arthritis
HRQoL: Health-related quality of life
MDA: Minimal disease activity
NSAID: Non-steroidal anti-inflammatory drug
OMERACT: Outcome Measures in Rheumatology
PsA: Psoriatic arthritis
PsAID-12: 12-item Psoriatic Arthritis Impact of Disease questionnaire
SD: Standard deviation
