# Supplementary material for: Results of a global, patient-based survey assessing the impact of psoriatic arthritis discussed in the context of the Psoriatic Arthritis Impact of Disease (PsAID) questionnaire
Source: Health Qual Life Outcomes. 2020 Jun 8;18:173. doi: 10.1186/s12955-020-01422-z (PMC7282161; doi:10.1186/s12955-020-01422-z)
Supplement: Supplementary file 1 — Additional file 1: Supplementary Table 1. Survey questions and responses. [file 12955_2020_1422_MOESM1_ESM.docx]

**Supplementary Table 1** Survey questions and responses

| **Survey question** | **Possible responses** |
| --- | --- |
| Q607. Length of time since diagnosis, years^a^  Q270. In what year were you born? Please enter your response as a four-digit number (e.g., 1977).  Q280. [HIDDEN QUESTION - FINAL AGE]  Q605. How old were you when you were first diagnosed with psoriatic arthritis by a healthcare professional? Your best estimate is fine. |  |
| Q630. Please indicate all prescription medications you are currently taking for psoriatic arthritis. Please select all that apply. | - Biologic treatment prescribed by a doctor, either injected or infused through an IV - Medication called “DMARDs”, which are prescribed by a doctor - Medication called “NSAIDs” or Cox-2 inhibitors, prescribed by a doctor for relief of pain and inflammation - Steroid medication (oral, topical, or injected) prescribed by a doctor to help control the symptoms of psoriatic arthritis - Another prescription medication for psoriatic arthritis - Not sure - I am not currently taking any prescription medications for psoriatic arthritis |
| Q631. How bad is your psoriatic arthritis today? | - Mild - Moderate - Severe |
| Q635. How would you describe your current overall health today? | - Excellent - Good - Fair - Poor |
| Q700. Have you done any of the following as a result of psoriatic arthritis? Please select all that apply. | - Experienced emotional distress - Stopped doing or participating in certain sports or recreational activities - Stopped doing or participating in social activities - Experienced social shame or disapproval - Taken a sick day from work - Decreased productivity at work - Taken medical leave from work - Went on permanent disability - Quit or been let go from a job - Switched jobs - Postponed having children - None of these |
| Q705. How much of a negative impact, if any, has psoriatic arthritis had on each of the following aspects of your life? Scale: no impact, slight impact, moderate impact, major impact. | - My level of physical activity - My ability to perform certain activities - My emotional/mental wellbeing - My productivity at work - My career path (e.g., industry/career I selected, career growth/ability to get promoted, etc.) - Romantic relationships or intimacy - Relationships with friends and family - My decision to start a family - My education |
| Q720. Which of the following symptoms, if any, have you experienced in the past 12 months related to psoriatic arthritis? Please select all that apply. | - Joint pain - Joint tenderness - Joint swelling - Stiffness - Inflammatory back pain (back pain/stiffness) - Tenderness or swelling of ligament/tendon that connects to the bone - commonly the heel or elbow (enthesitis) - Swollen or inflamed (“sausage”) fingers or toes (dactylitis) - Joint damage - Skin patches or plaques (e.g., flaking, redness, etc.) - Skin discomfort (e.g., itching, painful, bleeding, etc.) - Nail changes (e.g., pitting or small dents, separation from nail bed, etc.) - Unusual fatigue - Other - None, I have not experienced any symptoms of psoriatic arthritis in the past 12 months |
| Q725. You mentioned that you have experienced symptoms related to psoriatic arthritis in the past 12 months. Of these symptoms, which is the most bothersome? [asked only among patients who had experienced symptoms in the past 12 months]. | - Joint pain - Joint tenderness - Joint swelling - Stiffness - Inflammatory back pain (back pain/stiffness) - Tenderness or swelling of ligament/tendon that connects to the bone - commonly the heel or elbow (enthesitis) - Swollen or inflamed (“sausage”) fingers or toes (dactylitis) - Joint damage - Skin patches or plaques (e.g., flaking, redness, etc.) - Skin discomfort (e.g., itching, painful, bleeding, etc.) - Nail changes (e.g., pitting or small dents, separation from nail bed, etc.) - Unusual fatigue - Other - None of these symptoms are bothersome |
| Q1015. Which of the following symptoms do you still experience despite psoriatic arthritis treatment? Please select all that apply.  “I still experience...” [asked only among patients who were currently taking prescription medications for psoriatic arthritis]. | - Joint pain - Joint tenderness - Joint swelling - Stiffness - Inflammatory back pain (back pain/stiffness) - Tenderness or swelling of ligament/tendon that connects to the bone - commonly the heel or elbow (enthesitis) - Swollen or inflamed (“sausage”) fingers or toes (dactylitis) - Joint damage - Skin patches or plaques (e.g., flaking, redness, etc.) - Skin discomfort (e.g., itching, painful, bleeding, etc.) - Nail changes (e.g., pitting or small dents, separation from nail bed, etc.) - Unusual fatigue - Other - I do not still experience any symptoms |

^a^ Calculated by subtracting respondents’ answers to Q605 (How old were you when you were first diagnosed with psoriatic arthritis by a healthcare professional?) from their age (Q280; based on year of birth [Q270])

DMARD, disease-modifying antirheumatic drug; IV, intravenous; NSAID, non-steroidal anti-inflammatory drug
