# Supplementary material for: Results of a global, patient-based survey assessing the impact of psoriatic arthritis discussed in the context of the Psoriatic Arthritis Impact of Disease (PsAID) questionnaire
Source: Health Qual Life Outcomes. 2020 Jun 8;18:173. doi: 10.1186/s12955-020-01422-z (PMC7282161; doi:10.1186/s12955-020-01422-z)
Supplement: Supplementary file 2 — Additional file 2: Supplementary Table 2. Categorization of questions by domain of health. [file 12955_2020_1422_MOESM2_ESM.docx]

| **Domain of health** | **Question** |
| --- | --- |
| Pain | Q720, Q725, Q1015 |
| Fatigue | Q720, Q725, Q1015 |
| Skin | Q720, Q725, Q1015 |
| Work/Leisure | Q700, Q705 |
| Function | Q700, Q705 |
| Discomfort | Q720, Q1015 |
| Sleep |  |
| Coping | Q700, Q705 |
| Anxiety | Q700, Q705 |
| Embarrassment/Shame | Q700 |
| Social Participation | Q700 |
| Depression | Q700, Q705 |

**Supplementary Table 2** Categorization of questions by domain of health
