# Supplementary figures and images for: Results of a global, patient-based survey assessing the impact of psoriatic arthritis discussed in the context of the Psoriatic Arthritis Impact of Disease (PsAID) questionnaire
Source: Health Qual Life Outcomes. 2020 Jun 8;18:173. doi: 10.1186/s12955-020-01422-z (PMC7282161; doi:10.1186/s12955-020-01422-z)

Supplementary Figure 1

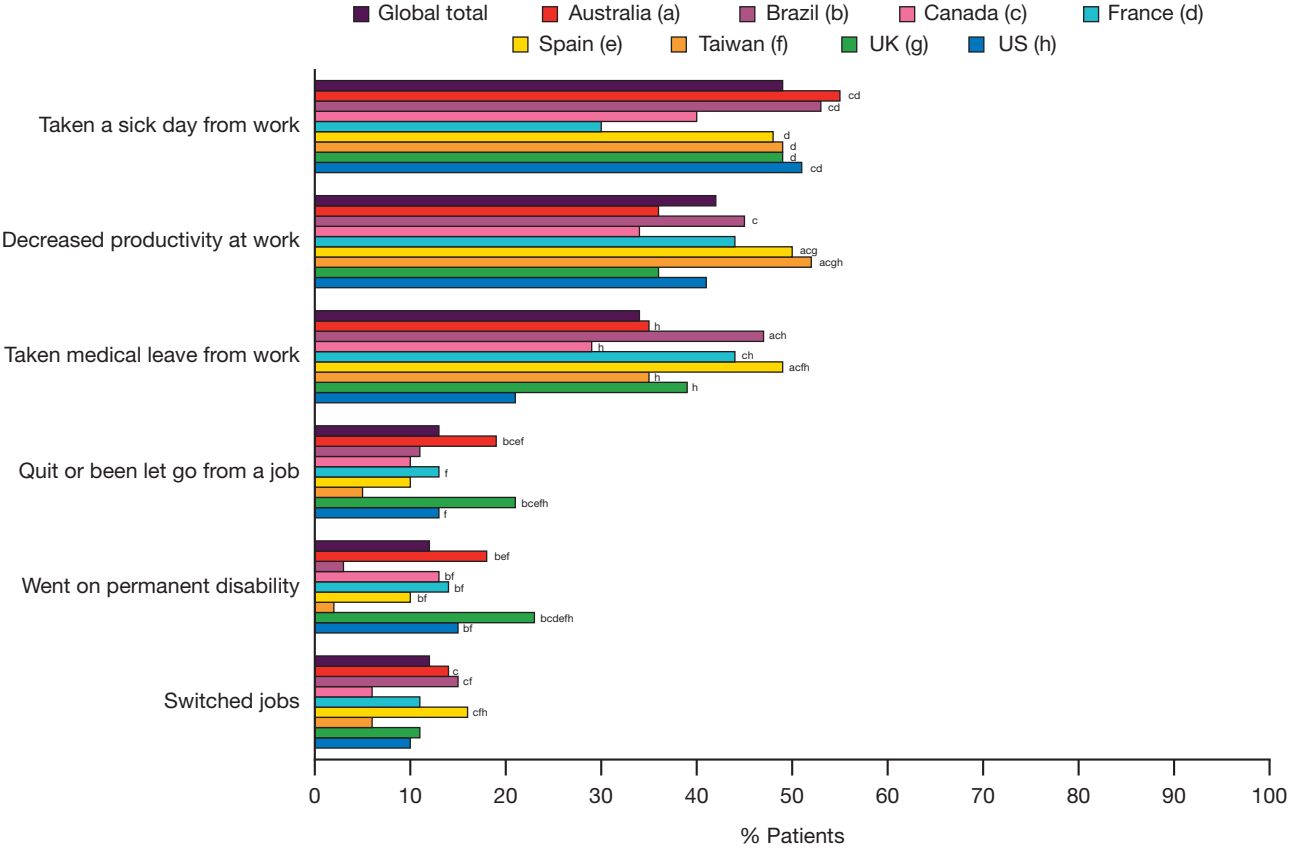

Supplement: Supplementary file 3 — Additional file 3: Supplementary Figure 1. Patient-reported impact of PsA on work. Data represent the percentage of responders using the weighted base of each country in Table 1. Data are reported in response to the question, “Have you done any of the following as a result of psoriatic arthritis? Please select all that apply.” Countries are represented by colored data bars. Significant differences (p < 0.05) between countries are designated by the letters following the bars: a = Australia; b = Brazil; c = Canada; d = France; e = Spain; f = Taiwan; g = UK; h = US. PsA, psoriatic arthritis. [file 12955_2020_1422_MOESM3_ESM.pdf]
